# Cross-Species Transcriptomic and Metabolomic Analysis Reveals Conserved and Divergent Fatty Acid Metabolic Regulatory Strategies During Mammalian Oocyte Maturation

**DOI:** 10.3390/ijms27010397

**Published:** 2025-12-30

**Authors:** Mostafa Elashry, Yassin Kassim, Bingjie Hu, Hao Sheng, Guangjun Xu, Hagar Elashry, Kun Zhang

**Affiliations:** 1 Key Laboratory of Dairy Cow Genetic Improvement and Milk Quality Research of Zhejiang Province, College of Animal Sciences, Zhejiang University, Room 301 E Building, 866 Yuhangtang Rd., Hangzhou 310058, China; elashry2000@mans.edu.eg (M.E.);; 2 Department of Animal Production, Faculty of Agriculture, Mansoura University, Mansoura 35516, Egypt; 3 Department of Animal and Poultry Production, Faculty of Agriculture, Minia University, El-Minya 61519, Egypt; 4 Nutrition and Clinical Nutrition Department, Faculty of Veterinary Medicine, Mansoura University, Mansoura 35516, Egypt

**Keywords:** oocyte, lipid, metabolomics, lipidomics, transcriptomics, meiosis

## Abstract

Mammalian oocyte maturation is a metabolically demanding process relying on lipid metabolism that supplies energy, structural substrates, and signaling mediators. However, a comprehensive cross-species understanding of the dynamic requirement for lipids during this process remains elusive, hindering the optimization of assisted reproductive technologies. Utilizing an integrated single-cell transcriptomic and targeted lipidomic approach, we mapped the metabolic landscape of bovine oocyte maturation. Our analysis uncovered a global transcriptional downregulation, with 3259 genes suppressed during the transition from the germinal vesicle (GV) to the metaphase II (MII) stage. This was particularly apparent in lipid catabolism pathways (e.g., for *ACAA1*), while mitochondrial energy production genes (*ATP6*) were upregulated. Lipidomics indicated a selective depletion of saturated fatty acids (SFAs; e.g., C16:0, C18:0) in MII oocytes, while monounsaturated (MUFAs) and polyunsaturated fatty acids (PUFAs) were preferentially retained. Integrated network analysis specified hexadecanoic acid (C16:0) as a central metabolic hub, which rewires its interactions from biosynthetic genes (*FASN*, *ELOVL6*) in GV oocytes to degradative enzymes (*ACADVL*, *HADH*) in MII oocytes. Expanding to a cross-species transcriptomic atlas, we identified a core set of 59 lipid metabolism genes conserved across bovine, mouse, and human oocytes. Despite this conservation, we discovered stark species-specific regulatory strategies: bovine and human oocytes significantly downregulated fatty acid degradation and elongation post-maturation, whereas murine oocytes maintain pathway activity, upregulating key regulators like *Acsl3*. Our work unveils an evolutionarily conserved core lipid metabolic program in mammalian oocytes that is adaptively tuned to meet species-specific physiological demands. Bovine and human oocytes prioritize catabolic flexibility, using SFAs for energy, while mouse oocytes maintain their anabolic capacity for membrane biosynthesis. These findings provide a transformative resource for the field, offering biomarkers for oocyte quality and a rationale for enhancing species-tailored lipid formulations to develop in vitro maturation systems and amend reproductive outcomes in both agriculture and medicine.

## 1. Introduction

Oocyte maturation is a pivotal process in female reproduction, comprising the transition from the germinal vesicle (GV) to the metaphase II (MII) stage, during which the oocyte attains the molecular and structural legitimacy prerequisite for fertilization and early embryogenesis in mammals [1,2]. This maturation process is supported by tightly controlled metabolic programs that regulate energy provision, the biosynthesis of macromolecules, and redox homeostasis [3,4]. Among these metabolic processes, lipid metabolism plays a particularly critical role, not only as a dense energy source but also as a supplier of structural components for membrane remodeling, precursors for signaling molecules, and modulators of intracellular signaling cascades [4,5,6].

The storage of lipids in oocytes occurs predominantly in the form of lipid droplets, whose abundance, composition, and mobilization exhibit variation across species and developmental stages [7,8]. During oocyte maturation, the balance between lipid anabolism (fatty acid biosynthesis and elongation) and catabolism must be precisely regulated to meet the changing energetic and structural requirements [4]. In bovine oocytes, the lipid content is comparatively higher than in rodents, reflecting a greater dependence on fatty acid oxidation (β-oxidation) for ATP generation during maturation and early embryonic cleavage [5,9]. This phenomenon contrasts with mouse oocytes, which exhibit a more substantial reliance on glycolytic and amino acid metabolism [10]. Such interspecies contrasts underscore the necessity for comparative analyses to distinguish both conserved and taxon-specific features of oocyte metabolic regulation in mammals.

Advances in single-cell RNA sequencing (scRNA-seq) have enabled the enhancement of oocyte transcriptomic resolution, facilitating the elucidation of the dynamic gene expression programs that drive meiotic progression [6,10]. When combined with targeted lipidomics, it is possible to integrate these datasets and establish a correlation between transcriptional regulation and metabolite levels. This approach can facilitate the identification of dynamic metabolic pathways and potential regulatory mechanisms [11,12]. Importantly, multi-omics approaches permit the analysis of metabolic networks at a systems level, qualifying the characterization of hub metabolites, co-regulated gene clusters, and stage-specific interaction modalities [11,12]. While studies of the transcriptome and lipidome have been published separately in oocytes of individual species, there remains a scarcity of integrated, cross-species datasets that hold both conserved and divergent metabolic designs.

Here, a multifaceted approach encompassing single-cell transcriptomics and targeted lipidomics was utilized to elucidate the intricate dynamics of lipid metabolic remodeling during bovine oocyte maturation. The integration of the bovine dataset with recently generated scRNA-seq profiles from mouse and human oocytes was further pursued to establish a cross-species transcriptomic atlas of lipid metabolism. The fundamental structure of oocyte lipid utilization was delineated by identifying a core set of lipid metabolism genes that are conserved across mammals. An evolutionary context for metabolic diversity was provided by identifying species-specific adaptations linked to energy requirements and the developmental tempo. The rewiring of metabolic networks at different stages was defined. Furthermore, we propose the utilization of specific molecules, including C16:0, as potential biomarkers for the assessment of oocyte quality in assisted reproductive technology (ART). The present research provides novel insights into the functional and evolutionary environment of lipid metabolism in oocytes.

## 2. Results

### 2.1. A Unified Multi-Omics Perspective on Lipid Metabolic Reprogramming During Bovine Oocyte Maturation

#### 2.1.1. Global Transcriptional Suppression and Maturation-Associated Gene Expression Changes

The analysis of the bovine oocyte transcriptomic landscape during IVM provides a foundational dataset for understanding the dynamic metabolic shifts that underpin meiotic competence acquisition. High-resolution scRNA-seq was performed on oocytes transitioning from the GV to the MII stage (Figure S1a), with a total of 28,779 genes profiled. The distinct separation of GV and MII oocytes in the t-SNE plot (Figure S1b) further confirmed the robustness and quality of the scRNA-seq data, validating that the observed gene expression patterns correspond to discrete developmental states. The analysis revealed a profound and highly significant change in gene expression, with 3407 differentially expressed genes (DEGs) identified between the two stages (Padj < 0.05, ∣log2 FC∣ > 1). This global change was overwhelmingly characterized by transcriptional suppression; a striking 3259 genes were downregulated during maturation, while only 148 genes showed increased expression (Figure S1c,d). This finding is not merely a consequence of maturation but appears to be a deliberate cellular strategy. The oocyte, poised for a state of meiotic arrest and subsequent transcriptional quiescence in the early embryo, reduces the energetic burden of transcription and translation by clearing a vast number of maternal transcripts.

The volcano plot and heatmap visualizations (Figure S1c,d) reinforced this dramatic shift, highlighting the significant suppression of genes related to lipid catabolism, such as the downregulation of acyl-CoA acyltransferase 1 (*ACAA1*; log2 FC < −2 and Padj < 1 × 10^−10^). This downregulation of key catabolic enzymes suggests that the oocyte’s focus is shifting away from active synthesis of these enzymes. In contrast, the mitochondrial ATP synthase gene, *ATP6*, was upregulated (log2 FC > 1.5, Padj < 0.01), indicating a strategic pivot toward direct energy production via oxidative phosphorylation (Figure S1c).

#### 2.1.2. Dynamic Regulation of Lipid Metabolism Gene Modules

**Figure 1:**
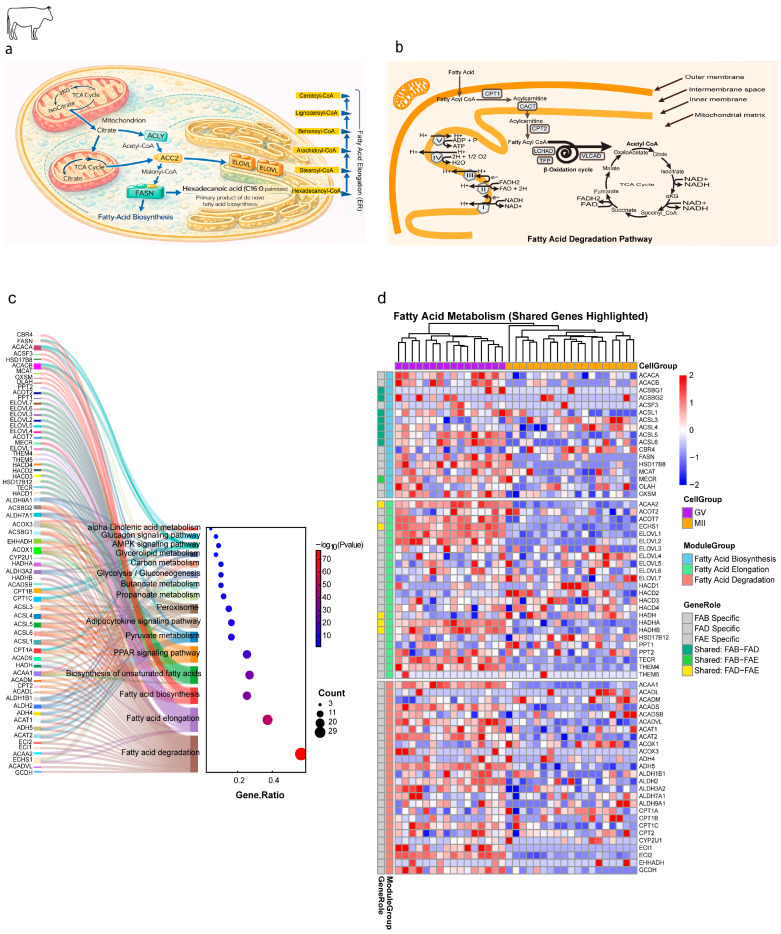
Remodeling of mitochondrial lipid metabolism pathways during bovine oocyte maturation. (**a**) De novo fatty acid biosynthesis in the cytosol generates hexadecanoic acid (palmitic acid; C16:0) as the central metabolic hub. Palmitoyl-CoA is subsequently elongated in the endoplasmic reticulum by the ELOVL enzyme family in sequential +2-carbon cycles, producing long- and very-long-chain fatty acyl-CoAs (C18–C26). (**b**) Fatty acid degradation (β-oxidation) and energy generation. Pathway highlights: (1) carnitine shuttle (*CPT1/2*), (2) β-oxidation cycle (*VLCAD*, *HADHA/B*), (3) TCA cycle and oxidative phosphorylation (ATP synthase). (**c**) Functional annotation of lipid metabolism genes. Left: Sankey diagram linking genes to pathways (Fatty Acid Biosynthesis [FAB], Elongation [FAE], Degradation [FAD]). Shared genes are color-coded. Right: Dot plot of enriched pathways (GeneRatio vs. −log_10_(*p*-value)). (**d**) Dynamic expression of lipid metabolism genes across maturation. Heatmap of genes grouped by pathway (FAB, FAE, FAD). Color scale: Z-score of expression (red: upregulated; blue: downregulated). Shared genes are annotated.

A more granular analysis of the core lipid metabolism gene modules (Figure 1a–d) revealed a sophisticated and asymmetric regulatory program. We focused on the precise regulation of three core mitochondrial lipid modules: FAB, FAE, and FAD (Figure 1a,b). Functional enrichment analysis of the expressed gene sets for these modules confirmed their significant involvement in central lipid regulatory pathways, including PPAR signaling, AMPK signaling, and adipocytokine signaling, highlighting the deep integration of these processes within the cellular metabolism (Figure 1c). The heatmap visualization of 67 expressed genes segregated the FAB, FAE, and FAD genes, demonstrating a predominant downregulatory trend during maturation (Figure 1d). 

**Figure 2:**
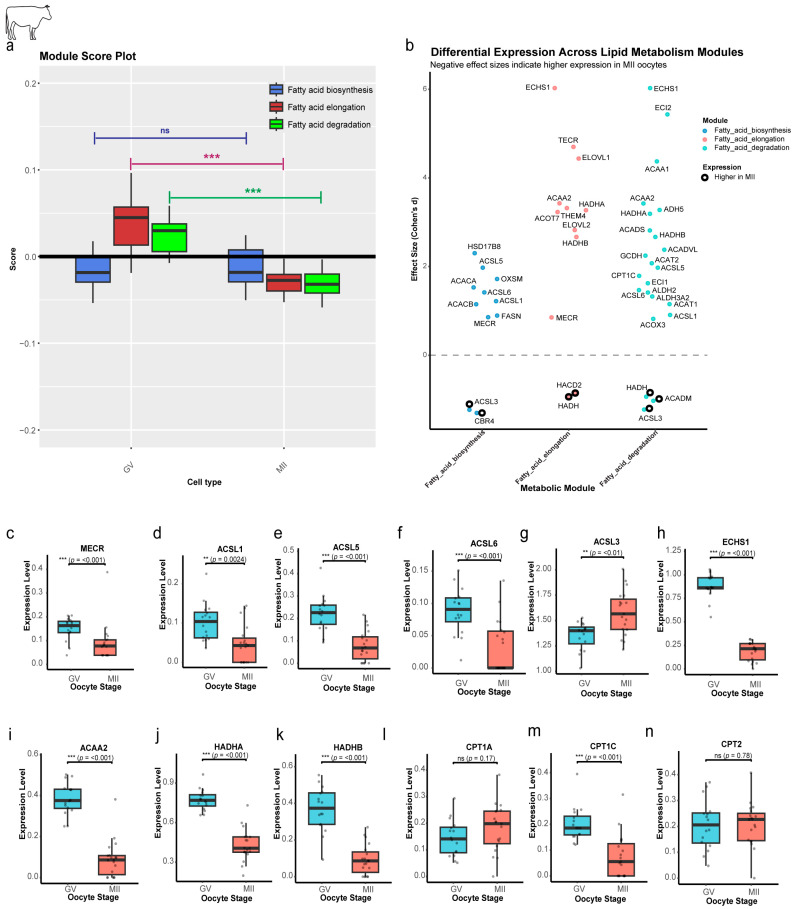
Stage-specific regulation of lipid metabolism genes and pathways in bovine oocytes. (**a**) Pathway activity scores by maturation stage. Module scores reveal reduced Elongation and Degradation (*p* < 0.01) in MII, while Biosynthesis remains stable (ns). (**b**) Effect-size analysis of module-specific genes. Dot plot of Cohen’s d effect size (GV vs. MII). Hollow circles: MII-enriched genes. Dashed line: null effect. (**c**–**k**) Stage-dependent expression of shared lipid metabolism genes. Box plots of genes shared between modules, showing significant downregulation from GV to MII. (**l**,**m**) *CPT1* gene expression dynamics. Only *CPT1C* is significantly reduced in MII. (**n**) Stable *CPT2* expression. *CPT2* (critical for acylcarnitine-to-acyl-CoA conversion) shows no significant change (ns). Statistical significance is indicated as ** *p* < 0.01, *** *p* < 0.001; ns, not significant (*p* > 0.05).

While the module score for FAB showed no significant change during maturation (*p* = 0.42), a dramatic and highly significant reduction was observed for both the FAE (*p* = 5.91 × 10^−9^) and FAD (*p* = 1.97 × 10^−9^) modules (Figure 2a). This selective downregulation suggests that the oocyte is not undergoing a generalized metabolic shutdown; rather, it is precisely remodeling its lipid-related machinery. Cohen’s d effect sizes quantify the degree of difference in gene expression between GV and MII stages, standardized to be interpretable across genes. Effect size is the magnitude of the difference between groups [13,14]. Most individual genes that have a significant large effect size (Padj < 0.05, d > 0.8) were enriched in the GV stage (31 genes), with only a few (5 genes) showing enrichment in the MII stage (Figure 2b), further demonstrating the overall trend of downregulation. Significantly large-effect genes that were shared among modules were enriched in the GV stage (Figure 2c–k). A notable exception to this pattern was *ACSL3* (Figure 2g), which was the only shared gene to be significantly enriched in the MII stage (Cohen’s d = −1.2), potentially due to its specific role in channeling fatty acids toward phospholipid synthesis. The carnitine palmitoyltransferase (*CPT*) gene family, which plays a key role in initiating the FAD pathway, also exhibited a complex regulatory pattern. Only *CPT1C* showed a significant reduction through maturation (Figure 2m), while *CPT1A* displayed a non-significant increase (Figure 2l), suggesting its potential role as the primary degradative isoform in mature oocytes. Meanwhile, *CPT2* maintained stable expression (*p* = 0.78) (Figure 2n). These findings suggest that the cell is not eliminating catabolism but, instead, relies on existing enzyme pools and selectively maintains specific isoforms.

The underlying gene co-expression networks also demonstrated a profound stage-specific transformation. At the GV stage, a strong positive correlation was observed among genes in the FAB, FAE, and FAD modules (Figure S2a), indicative of a coordinated and active anabolic state in which the cell is building and storing lipids. Furthermore, by the MII stage, the network had been markedly rewired. Correlations between gene pairs weakened, and new negative correlations emerged, most notably between *ACSL6* and *ACSL3* and between *ACSL6* and *ACAT1* (Figure S2b). The emergence of these negative interactions signals a shift in metabolic priorities. It suggests a more complex regulatory environment where the cell begins to prioritize one metabolic fate for a given lipid over another, thereby allocating resources in a highly controlled manner to meet the precise energetic and structural demands of meiotic progression.

#### 2.1.3. Targeted Lipidomics Uncovers Selective Catabolism of Saturated Fatty Acids

**Figure 3:**
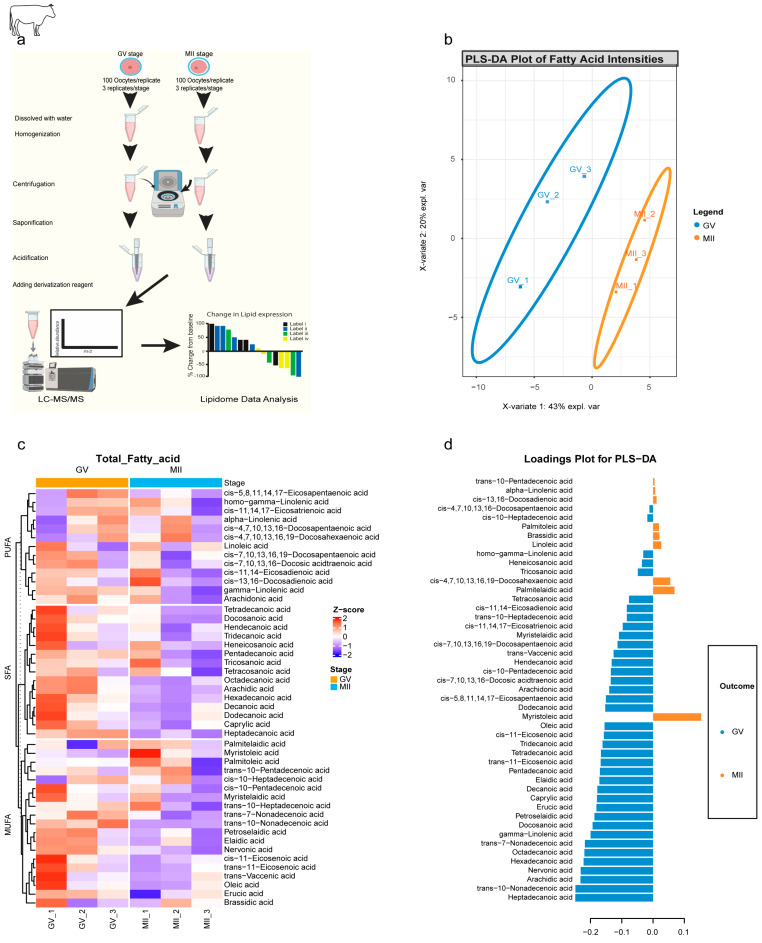
Stage-specific lipidomic signatures revealed by targeted metabolomics of bovine oocytes. (**a**) Workflow for targeted lipid metabolomics. Graphical abstract depicting (i) oocyte collection (GV/MII; 100 oocyte/replicate, 3 replicates/stage), (ii) methanol/MTBE extraction, (iii) saponification and derivatization, and (iv) LC-MS/MS analysis. (**b**) PLS-DA plot of fatty acid profiles. Clear separation of GV (blue) and MII (red) stages (X-variate 1: 43% explained variance; X-variate 2: 20%). Ellipses: 95% confidence intervals. (**c**) Clustered heatmap of 47 lipid metabolites. Hierarchical clustering of fatty acids across GV and MII replicates distributed into 3 main lipid categories: SFA, MUFA, and PUFA. Color scale: % change from baseline (red: upregulated; blue: downregulated). (**d**) PLS-DA loadings plot Metabolites driving stage separation: GV-associated (left blue; loading < −0.2): 38 metabolites. MII-associated (right orange; loading > 0.1): 9 metabolites.

To provide direct evidence of these metabolic shifts, targeted lipidomics was performed on GV and MII bovine oocytes using a robust LC-MS/MS workflow (Figure 3a). A Partial Least Squares–Discriminant Analysis (PLS-DA) plot (Figure 3b) revealed a clear and distinct separation between GV and MII oocytes, confirming the presence of stage-specific lipid signatures. A heatmap of lipid classes confirmed the uniform depletion of SFAs in MII oocytes, while MUFAs and PUFAs showed more heterogeneous, often stable, abundance (Figure 3c). The loadings plot identified 38 metabolites associated with GV oocytes, which comprised all SFAs (e.g., hexadecanoic acid, C16:0). In contrast, nine metabolites were associated with MII, predominantly MUFAs and PUFAs such as myristoleic acid (C14:1) and linoleic acid (C18:2) (Figure 3d). This finding points to a sophisticated and non-random metabolic prioritization.

**Figure 4:**
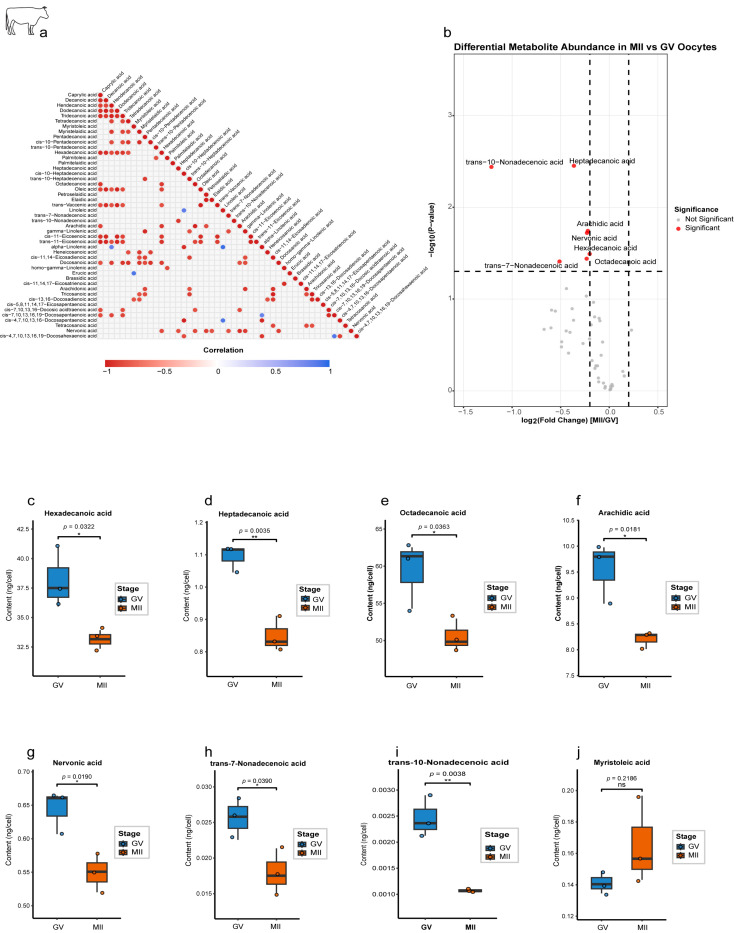
Stage-specific lipid metabolite dynamics during bovine oocyte maturation. (**a**) Correlation network of significantly associated lipid metabolites. Heatmap showing pairwise correlations among all detected lipids (*n* = 47). Only significant correlations (*p* < 0.05) are displayed, with 7 negative correlations (blue; range: −1 to 0) and positive correlations (red; 0 to 1). (**b**) Volcano plot of differential metabolite abundance. (**c**–**i**) Quantification of downregulated metabolites in MII oocytes. Box plots (ng/cell). (**j**) Myristoleic acid (C14:1) shows nonsignificant upregulation in MII. Statistical significance is indicated as * *p* < 0.05, ** *p* < 0.01; ns, not significant (*p* > 0.05).

Correlation network analysis of 47 lipid metabolites identified 134 significant interactions (|r| > 0.81, *p* < 0.05), with a strong predominance of positive co-regulation (127 edges), particularly among SFAs (e.g., C10:0 and C12:0; r = 0.99), suggesting their coordinated utilization (Figure 4a). We also observed critical negative correlations, most notably between the essential PUFAs linoleic acid (C18:2; ω-6) and α-linolenic acid (C18:3; ω-3) (r = −0.95, *p* = 0.003), suggesting potential competitive utilization of these structurally similar molecules for different metabolic fates, such as membrane incorporation or signaling pathways. A volcano plot identified seven significantly downregulated lipid metabolites in MII oocytes (Figure 4b), including key SFAs. Quantitative analysis revealed a pronounced depletion of SFAs during maturation. Hexadecanoic acid (C16:0) decreased by 15% (*p* < 0.001), octadecanoic acid (C18:0) decreased by 18% (*p* = 0.019), and arachidic acid (C20:0) decreased by 22% (*p* = 0.0035) (Figure 4c–f). Several MUFAs were also significantly affected (Figure 4g–i), including nervonic acid (C24:1; *p* = 0.039), trans-7-nonadecenoic acid (C19:1; *p* = 0.039), and trans-10-nonadecenoic acid (C19:1; *p* = 0.0038). However, other MUFAs did not show any significant change, such as myristoleic acid (C14:1), which showed a trend toward an increase but did not reach significance (*p* = 0.2186; Figure 4j).

**Figure 5:**
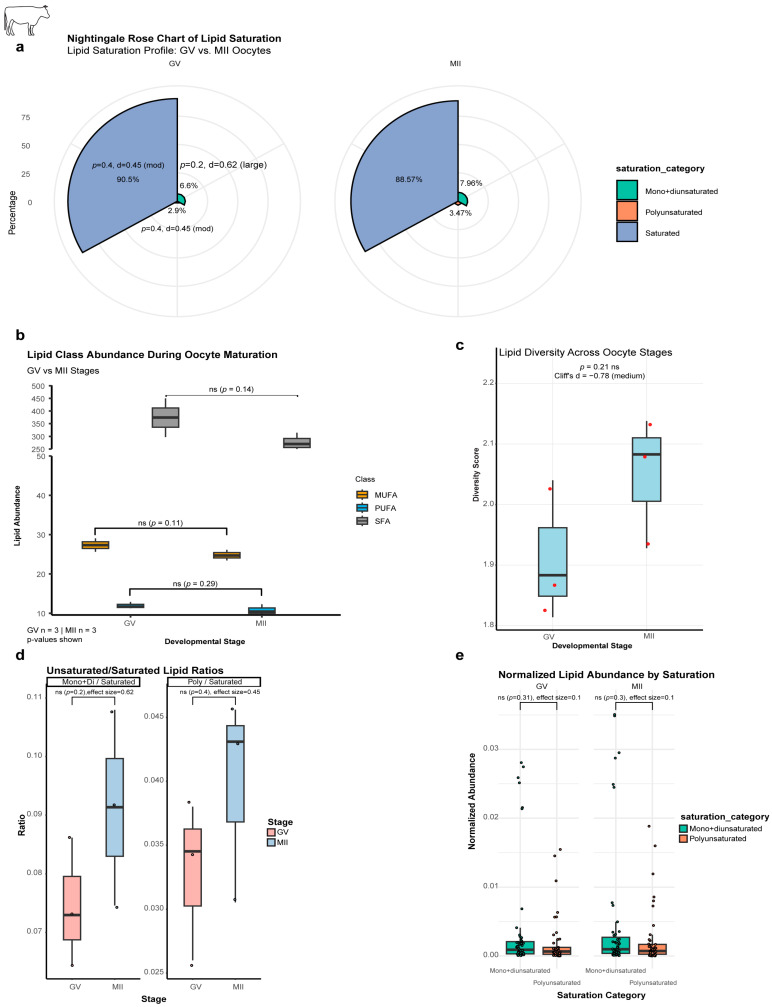
Lipid saturation remodeling during bovine oocyte maturation. (**a**) Nightingale Rose Chart of lipid saturation. Trends (%; non-significant): mono + diunsaturated (GV:6.6% → MII:7.9%); polyunsaturated (GV:2.9% → MII:3.5%); Saturated (GV:90.5% → MII:88.6%). (**b**) Lipid abundance by class (absolute quantities) exhibits global reductions (*p* > 0.05): SFA: 26% (GV:373.8 → MII:275.3); MUFA: 9% (GV:27.3 → MII:24.8); PUFA: 10% (GV:11.9 → MII:10.8). (**c**) Lipid diversity score: increased diversity in MII (GV:1.91 → MII:2.00; Cliff’s δ = −0.78 [moderate effect]). (**d**) Unsaturated/Saturated ratios; non-significant increases: mono+di/saturated: 22% (GV:0.075 → MII:0.091); poly/saturated: 21% (GV:0.033 → MII:0.040). (**e**) Normalized lipid abundance; non-significant trends: mono+di (GV:0.004 → MII:0.005); poly (GV:0.002 → MII:0.002). ns, not significant (*p* > 0.05).

The proportion of SFAs decreased from 90.5% to 88.6%, while MUFAs and PUFAs increased from 6.6% to 8% and 2.9% to 3.5%, respectively (Table 1 and Figure 5a). While absolute quantities of total SFAs, MUFAs, and PUFAs showed non-significant decreasing trends (26.3%, 9.4%, and 9.9%, respectively; *p* > 0.05) (Figure 5b), analysis of proportional composition and ratios revealed biologically relevant shifts. Furthermore, the lipid diversity score increased from GV to MII, supported by a moderate effect size (Cliff’s δ = −0.78) (Figure 5c). These shifts resulted in a 22.5% increase in the MUFA/SFA ratio (effect size δ = 0.62) and a 21% increase in the PUFA/SFA ratio (effect size δ = 0.45) (Figure 5d). Normalized abundance trends were consistent but non-significant (Figure 5e). This collective pattern suggests that the oocyte preferentially consumes SFAs for energy while retaining unsaturated lipids to preserve membrane fluidity and to support the extensive membrane remodeling required during meiotic progression.

#### 2.1.4. Integrative Network Analysis: Hexadecanoic Acid (C16:0) as a Dynamic Metabolic Hub

The integration of transcriptomic and lipidomic data, through correlation network analysis, provided the most compelling evidence of a coordinated metabolic switch. This analysis pinpointed hexadecanoic acid (C16:0) as a central and dynamically regulated hub (Figures S3 and S4). At the GV stage, the correlation heatmap (Figure S3a) demonstrated strong co-regulation between 47 lipid metabolites categorized into SFA, MUFA [*cis/trans*], and PUFA [ω-3/ω-6] and lipid-module genes involved in FAB (*FASN*, *ACACA*), FAE (*ELOVL1/2/6*), and FAD (*ACAA2*, *ACADVL*). C16:0 was a highly correlated node in a complex network (Figure S3b), showing strong positive associations with both FAB genes like fatty acid synthase (*FASN*; r = 1.00, *p* = 0.013) and acetyl-CoA carboxylase α (*ACACA*; r = 1.00, *p* = 0.001), and elongation genes such as *ELOVL2* and *ELOVL6*. The correlation with degradation enzymes like *ACAA1* and *ACADM* further highlights a state of balanced metabolic flux, where synthesis, elongation, and degradation are all active and coordinated. This anabolic phase is crucial for building the energy reserves that the oocyte will rely on during its final maturation, consistent with its role as an end-product of mitochondrial elongation and a substrate for both storage and energy production [3,15].

By the MII stage, the network was profoundly rewired (Figure S4a), reflecting a fundamental metabolic pivot. The correlations between C16:0 and its biosynthetic genes were dramatically weakened (*FASN*; r = 0.02), while strong new positive correlations emerged with degradation genes, such as acyl-CoA dehydrogenase very long chain (*ACADVL*) and hydroxyacyl-CoA dehydrogenase trifunctional multienzyme complex subunit α (*HADH*). This shift directly links the observed lipidomic depletion of C16:0 to a coordinated gene expression program aimed at its catabolism. A striking example of this regulatory rewiring was the gene *MECR*, encoding a protein that catalyzes the last step in mitochondrial fatty acid synthesis [16], which switched from a strong positive correlation in GV (r = 1.00, *p* = 0.02) to a negative one in MII (r = −1.00, *p* = 0.01). This demonstrates a specific, stage-dependent regulatory switch. Furthermore, the analysis revealed a functional partitioning among acyl-CoA synthetase family members in the MII stage. *ACSL1* displayed a strong inverse relationship with omega-3 PUFAs (r = −0.982, *p* = 0.120), suggesting its potential role in diverting these long-chain fatty acids away from catabolism and toward crucial structural roles in membrane remodeling. In contrast, *ACSL3* showed a robust positive correlation with C16:0 (r = 0.912, *p* = 0.270), indicating its pivotal role in the activation and subsequent degradation of SFAs for energy production via β-oxidation to meet the high energetic demands of meiotic progression (Figure S4b).

### 2.2. Comparative Transcriptomic Analysis of Lipid Metabolism in Mouse and Human Oocytes

#### 2.2.1. Transcriptomic Dynamics in Mouse Oocytes Exhibit Distinct Regulatory Patterns

**Figure 6:**
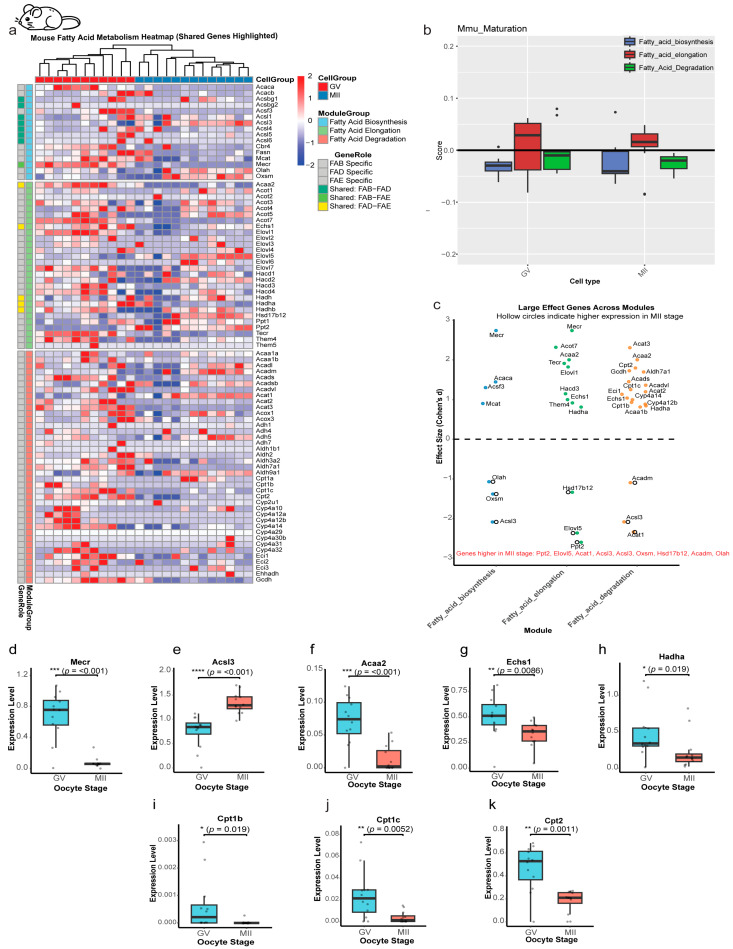
Stage-specific regulation of lipid metabolism genes in mouse oocytes. (**a**) Heatmap of lipid metabolism gene expression. Dynamic changes across GV and MII stages, categorized by: Fatty Acid Biosynthesis (FAB), Elongation (FAE), and Degradation (FAD) or shared between 2 modules. Color scale: Z-score of expression (red: upregulated; blue: downregulated). Shared genes are annotated. (**b**) Pathway activity scores across maturation stages. Module scores reveal that all three modules (Biosynthesis, Elongation, Degradation) show no significant differences between the GV and MII stages in mouse oocytes (*p* > 0.05 for all comparisons). (**c**) Effect-size analysis of module-specific genes. Cohen’s d values (GV vs. MII) indicating GV-enriched (highlighted) or MII-enriched (hollow circle) genes. (**d**–**h**) Stage-dependent expression of shared genes. Box plots illustrate the expression patterns of significantly shared genes across lipid metabolism modules, revealing a general downregulation from GV to MII oocytes. In contrast, *Acsl3* shows significant upregulation (****, *p* < 0.001). (**i**,**j**) *Cpt1* gene expression dynamics. Both *Cpt1b*, *c* are significantly reduced at the MII stage compared with GV. (**k**) *Cpt2*, a key enzyme mediating acylcarnitine-to-acyl-CoA conversion, exhibits significant downregulation during oocyte maturation. Statistical significance is indicated as * *p* < 0.05, ** *p* < 0.01, *** *p* < 0.001; ns, not significant (*p* > 0.05).

The single-cell transcriptomic analysis of mouse oocytes revealed a lipid metabolic reprogramming strategy that contrasts starkly with the bovine model (Figure 6 and Figure S5). Clear separation between the developmental stages was confirmed by t-SNE visualization (Figure S5a). A total of 5670 DEGs were identified between the GV and MII stages (4338 downregulated, 1332 upregulated; Padj < 0.05; Figure S5b,c). Functional annotation revealed significant enrichment in lipid metabolic pathways (Figure S5d).

Heatmap analysis demonstrated dynamic expression patterns of lipid metabolism genes across the GV and MII stages (Figure 6a) in mouse oocytes, which showed a different pattern of expression change compared to that in bovine oocytes. However, despite these gene-level changes, the pathway activity module scores for FAB, FAE, and FAD remained stable throughout maturation (*p* > 0.05; Figure 6b), a finding that fundamentally differentiates the mouse from the bovine and human models. This stability at the pathway level masks significant changes at the individual gene level. Cross-module analysis identified 33 genes with a large effect size. Notably, only 8 of these were upregulated in the MII stage, while the remaining 25 were enriched in GV oocytes (Figure 6c). Of all the shared genes among the lipid modules under study (Figure 6d–h), *Acsl3* was the only one to show significant upregulation in MII (Cohen’s d = −2.1, *p* < 0.001; Figure 6e), while a uniform downregulation was observed across all three *CPT* family genes (*Cpt1b*, *Cpt1c*, and *Cpt2*) (d = +1.8 to +1.3, *p* < 0.01; Figure 6i–k). This contrasts with the bovine pattern, where only *CPT1C* was significantly suppressed.

Correlation network analysis revealed stage-specific coordination: GV oocytes exhibited exclusively positive correlations among lipid genes (Figure S2c), while MII oocytes showed a more complex network, including two significant negative correlations (*Acsl6* and *Acsl3*; *Acsl6* and *Acat1*) amidst otherwise positive interactions (Figure S2d), which suggests an increasing complexity in metabolic regulation as the oocyte prepares for fertilization. This stable pathway activity, coupled with specific gene-level regulation, suggests that mouse oocytes, which have a high reliance on glycolysis, may maintain their lipid metabolic machinery in a ready state, finely tuning its activity through a different set of regulatory mechanisms.

#### 2.2.2. Human Oocytes Undergo Significant Downregulation of Core Metabolic Pathways

**Figure 7:**
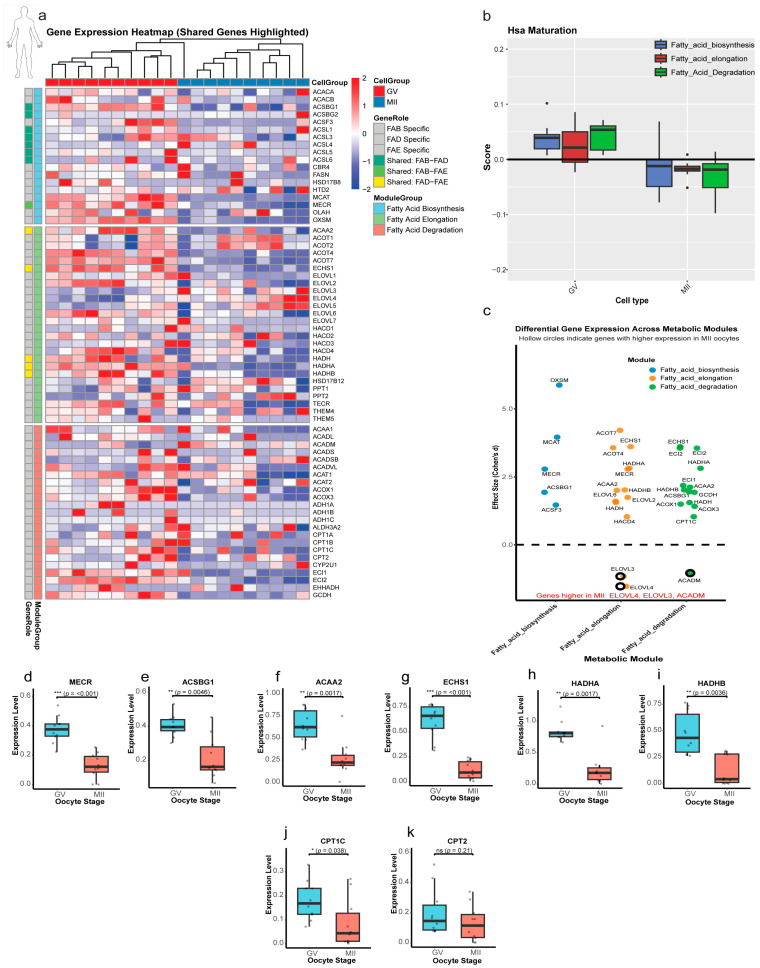
Stage-specific regulation of lipid metabolism genes in human oocytes. (**a**) Heatmap of lipid metabolism gene expression. Z-score normalized expression of genes grouped by Fatty Acid Biosynthesis (FAB), Elongation (FAE), and Degradation (FAD). Shared genes are highlighted at the module interfaces. (**b**) Pathway activity scores. Module scores in human oocytes showed a reduction in the 3 lipid modules from GV to MII. (**c**) Effect-size analysis (Cohen’s d). GV-enriched: (d > 0; filled in circle); MII-enriched: (d < 0; hollow circle). (**d**–**i**) Shared gene dynamics. Box plots confirm downregulation in all of them through the transition from the GV to MII stage. (**j**) *CPT1C* expression, showing a significant reduction in MII. (**k**) *CPT2* stability. No change (ns; *p* = 0.21), maintaining basal β-oxidation capacity. Statistical significance is indicated as * *p* < 0.05, ** *p* < 0.01, *** *p* < 0.001; ns, not significant (*p* > 0.05).

The analysis of human oocytes revealed a metabolic strategy that more closely parallels the bovine than the murine model (Figure 7 and Figure S6). The clear separation between developmental stages was confirmed by t-SNE visualization (Figure S6a). A total of 4911 DEGs were identified during the GV to MII transition, with a more balanced distribution (2827 downregulated, 2084 upregulated; Padj < 0.05; Figure S6b,c). Functional annotation demonstrated pronounced enrichment for the FAD (*p* = 2.6 × 10^−68^), FAE, and FAB pathways (Figure S6d).

Heatmap analysis demonstrated dynamic expression patterns of lipid metabolism genes across then GV and MII stages in human oocytes (Figure 7a), which showed a predominant downregulatory trend during maturation nearly similar to that in bovine oocytes. Module score analysis revealed a coordinated downregulation of lipid metabolic pathways in human oocytes, mirroring the bovine pattern. The FAB and FAE modules were significantly reduced in MII (*p* = 0.004 and 7.53 × 10^−2^, respectively), while the degradation module showed the most pronounced decrease (*p* = 7.6 × 10^−5^; Figure 7b). At the single-gene level, distinct regulatory patterns were observed. From 24 significant genes with large effects, only 3 genes (*ACADM, ELOVL3,* and *ELOVL4*) were upregulated in the MII stage, while other genes were upregulated in the GV stage. Key regulatory genes displayed distinct dynamics (Figure 7c): *OXSM* (FAB; d = +5.9, *p* < 0.001) and *ACOT7* (FAE; d = +4.2, *p* < 0.001) were strongly GV-enriched, while *ELOVL4* (FAE; d = −1.5, *p* = 0.002) showed MII-specific upregulation. All significant shared genes with large effect sizes showed significant downregulation from GV to MII (Figure 7d–i). The *CPT* gene family again demonstrated selective regulation, with *CPT1C* being downregulated in MII (*p* = 0.038; Figure 7j) while *CPT2* expression remained stable (*p* = 0.21; Figure 7k), echoing the bovine pattern and suggesting selective modulation of mitochondrial β-oxidation capacity.

As with the other two species, correlation network analysis revealed stage-dependent rewiring. GV oocytes showed predominantly positive correlations with one exception (*ACADS-CYP2U1*) (Figure S2e), while MII oocytes developed a more complex network with five significant negative correlations, most involving *CPT2* (e.g., *ACADM-CPT2, HADHA-CPT2*) and others like *ACAA2-ELOVL3* (Figure S2f), signaling a complex regulatory rewiring.

### 2.3. Evolutionary Conservation and Species-Specific Diversification

A comparative analysis of lipid metabolism genes across bovine, mouse, and human oocytes revealed a fundamental evolutionary framework (Figure S7). A core set of 59 genes was identified as being conserved across all three species (Figure S7a), representing the essential components of the FAB, FAE, and FAD pathways. This conserved toolkit included critical regulators such as *ACSL1*, *ELOVL1*, and *CPT2*, underscoring the deep evolutionary importance of these metabolic processes for successful oocyte maturation. The FAB module demonstrated the highest degree of conservation, with 16 genes shared across all species (Figure S7b), highlighting the fundamental importance of anabolism for preparing the oocyte. Human oocytes uniquely expressed *HTD2*, while *HSD17B8* was specifically shared between bovine and human oocytes. The FAE module maintained 25 universally conserved genes, with the mouse oocyte exhibiting two unique elongases (*ACOT3, ACOT5*) and sharing two others with human oocytes (*ACOT1, ACOT4*; Figure S7c). However, this conservation is not absolute. The FAD module showed the greatest species-specific variation (Figure S7d), suggesting that the precise strategies for lipid catabolism are more adaptable and have been uniquely tailored to meet species-specific physiological demands. For example, the mouse oocyte possesses 14 unique degradation-related genes (primarily Cyp4a family members), while human oocytes express three specific alcohol dehydrogenases (*ADH1A, ADH1B*, and *ADH1C*).

Notably, bovine oocytes lacked species-specific genes, with all expressed genes being shared with at least one other species. This core conservation underscores the essential nature of these metabolic processes, while the species-specific variations highlight adaptive evolutionary tuning. This result suggests that while all mammals inherited a basic genetic toolkit for lipid metabolism, the specific pathways and regulatory mechanisms for catabolism have diversified to suit differing reproductive strategies, such as variations in developmental tempo, oocyte size, and energy storage capacity.

## 3. Discussion

Our multi-omics investigation has unraveled some of the essential rules governing lipid metabolic regulation during oocyte maturation across three mammalian species while revealing crucial species-specific adaptations that reflect discrete evolutionary strategies. The integration of single-cell transcriptomics and targeted lipidomics offers unprecedented accuracy regarding the dynamic metabolic alterations implying meiotic competence acquisition, providing new insights into both conserved mechanisms and taxon-specific innovations.

The detection of 59 core lipid metabolism genes conserved across bovine, mouse, and human oocytes (Figure S7a) illustrates a landmark finding that emphasizes the substantial value of lipid homeostasis for efficacious oocyte maturation. This conserved gene set encompasses all major pathways, including FAB (*ACACA*, *FASN*), FAE (*ELOVL1-7*), and FAD (*CPT*, *ACADVL*) [17], forming an evolutionary toolkit that has been maintained through approximately 80 million years of mammalian divergence [18,19]. These results align with recent comparative genomic studies demonstrating the strong purifying selection of metabolic genes in mammalian germlines [20]. However, our temporal analysis revealed striking species differences in pathway regulation. While bovine and human oocytes showed significant downregulation of the FAD and FAE modules during maturation (Figure 2a and Figure 7b), mouse oocytes maintained stable pathway activity (Figure 6b). This regulatory divergence may reflect fundamental differences in energy substrate utilization, with murine oocytes relying more heavily on glycolytic flux [10] compared with the greater β-oxidation dependence observed in bovine and human oocytes. The stability of lipid pathway activity in mouse oocytes, despite meiotic progression, suggests the existence of compensatory mechanisms that maintain metabolic homeostasis, possibly through enhanced glucose utilization, as reported in murine COCs [21].

Our integrated transcriptomic–metabolomic analysis in bovine oocytes revealed sophisticated temporal coordination between gene expression patterns and lipid metabolite profiles. The strong positive correlations observed between lipogenic genes (*FASN*, *ELOVL6*) and hexadecanoic acid (C16:0) in GV oocytes (Figure S3b) suggest active de novo lipogenesis during early maturation, proportional with ultrastructural studies elucidating lipid droplet accumulation in pre-MII oocytes [6]. This anabolic phase seems critical for constructing energy reserves, as suppression of FAB during early maturation significantly decreases developmental competence in bovine oocytes [5]. The subsequent metabolic alteration toward C16:0-*ACADVL*/*HADH* correlations in MII oocytes (Figure S4b) reflects the well-documented transformation to oxidative phosphorylation in mature oocytes [22], with our lipidomic data providing novel evidence that SFAs are available as favored substrates for this process. The selective depletion of C16:0-C20:0 SFAs and C24:1 VLCFAs in MII oocytes (Figure 4c–i) likely reflects their preferential oxidation, as SFAs are more readily catabolized than unsaturated species due to their simpler molecular structure [23]. This finding aligns with our observation of maintained *CPT1A* expression (Figure 2l), which encodes the rate-limiting enzyme for mitochondrial fatty acid import. The concurrent retention of MUFAs such as myristoleic acid (C14:1; Figure 4j) may preserve membrane fluidity during meiotic progression, consistent with reports that MUFA-enriched diets improve oocyte quality in cattle [24]. Interestingly, the negative correlations between essential PUFAs (linoleic vs. α-linolenic acid; Figure 4a) suggest competitive utilization of these structurally similar molecules, potentially reflecting differential requirements for membrane incorporation versus energy production [25].

Several genes emerged as key regulators of taxon-specific metabolic strategies. In bovine oocytes, the selective downregulation of *CPT1C* (Figure 2m) suggests isoform-specific control of β-oxidation, potentially redirecting lipid flux toward somatic *CPT1A*-mediated β-oxidation [26,27]. This finding has particular relevance for improving bovine IVM systems, as the *CPT1* isoform balance may influence developmental competence [9]. Mouse oocytes displayed unique upregulation of *Acsl3* (Figure 6e), which preferentially activates C18-22 PUFAs for phospholipid synthesis [7,28], possibly reflecting the high membrane turnover demands of rapid murine embryogenesis. Human oocytes exhibited a distinctive induction of *ELOVL4* (Figure 7c), which elongates C26+ fatty acids critical for synaptic membrane formation [29,30], indicating preparation for the extended preimplantation period characteristic of human development. Universal downregulation of the divergent CPT family in mouse (*Cpt1b/c/2*; Figure 6i–k) versus selective *CPT1C* suppression in bovine/human highlights the evolutionary adaptations to differing energy demands based on oocyte size (approximately 80 µm in mice; 120–130 µm in large animals, including humans) and developmental tempo [31]. These findings substantially expand upon previous single-species studies by revealing how conserved metabolic pathways have been uniquely adapted across mammalian taxa.

The temporal regulation of the conserved lipid metabolic toolkit was highly species-specific, and these patterns correlated with known physiological differences, including established variations in the duration of in vitro oocyte maturation. Bovine oocytes in our study underwent IVM for 22–24 h (Section 4.1), and human IVM typically requires a longer period than murine maturation under standard culture conditions (24–30 h) [32,33]. Correspondingly, the large lipid-rich bovine and human oocytes downregulated anabolic and catabolic genes (FAE, FAD) during maturation, coinciding with a selective depletion of SFAs in the bovine model. This gradual metabolic downregulation may reflect a strategy for managing lipid reserves over extended developmental timelines. Conversely, mouse oocytes, which complete their maturation more rapidly in vitro (~12–16 h) [34,35,36], maintained stable pathway activity while uniquely upregulating *Acsl3*, an activator of long-chain PUFAs. This strategy may support rapid biosynthesis during a shorter maturation window. The specific induction of *ELOVL4* in human oocytes was another key point of divergence. These regulatory differences suggest that the conserved metabolic network is tuned to species-specific energetic and structural demands, with the duration of meiotic progression being a critical parameter shaping lipid strategies.

Our results have several important implications for ART. First, the species-specific lipid depletion patterns suggest that IVM systems may require tailored lipid supplementation, such as MUFA-enriched media for bovine/human oocytes versus balanced formulations for mice. Second, the conserved correlation between *ACSL3* and C16:0 could serve as a cross-species biomarker for lipid metabolic competence. Third, species-specific genes like human *HTD2* represent promising targets for investigating lipid-related infertility.

While our integrated multi-omics approach provides a systems-level view of lipid metabolic reprogramming, certain limitations should be noted. First, our study primarily utilizes an in vitro maturation (IVM) model. Although IVM is a standard and controlled system, it may not fully recapitulate the dynamic follicular microenvironment in vivo, particularly regarding paracrine signaling from cumulus cells and the influence of follicular fluid metabolites [37,38,39]. Second, our cross-species analysis relies on publicly available transcriptomic datasets generated by different laboratories using potentially varied protocols; while we performed rigorous normalization, batch effects cannot be entirely ruled out. Third, our targeted lipidomics quantified fatty acids but did not cover other critical lipid classes (e.g., phospholipids, sphingolipids) that are essential for membrane architecture and signaling. Fourth, our correlation-based network analyses, while insightful, indicate associations rather than direct causal relationships or metabolic fluxes. Fifth, we acknowledge that the lipidomics sample size (*n* = 3 biological replicates) limits the statistical power and increases the sensitivity to individual data points. To address this limitation, we complemented—and where appropriate prioritized—effect-size analysis (Cliff’s δ) over a sole reliance on *p*-values. Effect sizes are less dependent on sample size and provide a standardized estimate of biological magnitude [13]. Importantly, these trends were independently corroborated by our higher-n transcriptomic dataset, providing orthogonal multi-omics validation and strengthening confidence in the biological relevance of the results despite the limited lipidomics sample size.

These limitations define clear avenues for future research. Subsequent studies should (1) incorporate single-cell or spatial lipidomics to resolve lipid heterogeneity within oocyte subpopulations and between ooplasmic compartments [11]; (2) employ stable isotope tracing (metabolic flux analysis) to quantitatively trace the fate of specific fatty acids and to measure pathway activities in real time [12]; (3) perform functional validation via the genetic (e.g., siRNA) or pharmacological modulation of key regulators like *ACSL3* or *CPT1C* to establish their causal roles in determining oocyte quality; (4) design cross-species embryo-transfer experiments using lipid-modulated oocytes to directly assess the developmental outcomes of the metabolic strategies we identified; and (5) expand the evolutionary comparison to include additional mammalian lineages (e.g., porcine, non-human primate) to better resolve the adaptive landscape of oocyte metabolism. Finally, the functional importance of the stage-specific negative gene–metabolite correlations we observed in MII oocytes warrants detailed inspection, as these may signify critical regulatory switches or competitive substrate utilization at the culmination of maturation.

## 4. Materials and Methods

### 4.1. Bovine Oocyte In Vitro Maturation and Collection

The IVM method was performed in accordance with previously documented protocols [40,41]. This work was conducted in compliance with the ethics requirements of the Ethical Review Committee for Experimental Animal Welfare, Zhejiang University (Approval Code: 29850; Approval Date: 26 August 2024). In summary, ovaries from dairy cows were procured from a local slaughterhouse and transported to the laboratory in a saline solution supplemented with 200 IU/mL penicillin (P7794; Sigma, St. Louis, MO, USA) and 200 IU/mL streptomycin (S1277; Sigma, St. Louis, MO, USA) at temperatures between 30 and 35 °C within an 8–10 h period. After an extensive washing procedure consisting of three saline rinsing cycles, cumulus–oocyte complexes (COCs) with a minimum of three layers of cumulus cells (CCs) were harvested from 3 to 8 mm follicles located at the ovarian surface.

Subsequently, COCs exhibiting uniformly granulated cytoplasm and a minimum of three layers of CCs were transferred to an IVM medium consisting of Medium-199 (M4530) supplemented with 10% fetal bovine serum (FBS; Gibco-BRL, Grand Island, NY, USA), 1 IU/mL follicle-stimulating hormone (Sansheng Biological Technology, Ningbo, China), 0.1 IU/mL luteinizing hormone (Solarbio, Beijing, China), 1 mM sodium pyruvate (Thermo Fisher Scientific, Waltham, MA, USA), 2.5 mM GlutaMAX (Thermo Fisher Scientific, Waltham, MA, USA), and 10 μg/mL gentamicin. The incubation occurred at 38.5 °C in an environment with 5% CO_2_ and humidified air for 22 to 24 h. To obtain GV-stage oocytes, COCs were denuded immediately post-retrieval (0 h of IVM) by putting them into warmed medium including 0.3% (*w*/*v*) hyaluronidase (H3506; Sigma, St. Louis, MO, USA) at 38.5 °C for 5 min. For MII-stage oocytes, COCs were denuded after 24 h of maturation, and the extrusion of the first polar body was employed as a marker of nuclear maturation.

### 4.2. Single-Cell RNA-Seq Analysis

#### Dataset Assembly

A total of three distinct single-cell RNA-seq datasets were gathered and stage-matched to provide a collection of transcriptome data in pre- and post-maturation stages for three mammalian species. The datasets were analyzed in R Studio using R software version 4.2.2 (www.r-project.org). The Seurat v‘5.0.3’ platform was utilized for the analysis of single-cell RNA-seq data. t-Distributed Stochastic Neighbor Embedding (t-SNE) utilizing Seurat was performed to investigate clusters and to visualize the quality of the employed samples. Single datasets, as indicated by the t-SNE plots in Figures S1b, S5a, and S6a were log-normalized prior to the execution of any analysis. The analysis was conducted utilizing single cell datasets from GV to MII. Mus musculus (mouse) and Homo sapiens (human) datasets [42] were downloaded from Gene Expression Omnibus (GEO) using accession number GSE197578, while the Bos taurus (bovine) dataset was obtained from our laboratory (GSE304792). The procedures of bovine oocyte maturation, single-cell isolation, and library construction were accomplished according to a previously established protocol [43].

### 4.3. Compilation and Curation of Lipid Metabolism Gene Sets

Gene sets for Fatty Acid Biosynthesis (FAB), Elongation (FAE), and Degradation (FAD) were constructed from the Kyoto Encyclopedia of Genes and Genomes (KEGG) database using R. The particular KEGG pathway maps obtained were map00061 (FAB), map00062 (FAE), and map00071 (FAD) for the species Bos taurus (bta), Mus musculus (mmu), and Homo sapiens (hsa). The genes contained within these specified KEGG modules were then cross-referenced with our dataset’s expression matrix. Only genes with non-zero counts in at least one sample were kept for later module score calculation. The expression distribution of these filtered gene sets was visualized employing violin plots.

### 4.4. Module Score Analysis

Module scores for each pathway (FAB, FAE, FAD) were then calculated for every single cell using the AddModuleScore function in Seurat v‘5.0.3’ platform. This algorithm computes the average expression of the gene set and subtracts the average expression of a control gene set, which is formed by randomly selecting genes from bins of genes with similar average expression levels through the dataset. Accordingly, a positive module score suggests that the pathway’s genes are expressed more highly in a cell than a random background set of genes. Default parameters were utilized for this computation [10]. The expression of key genes related with these modules in cow, mouse, and human datasets was estimated using Seurat v‘5.0.3’ log-normalization and scaling, and significant differences were computed using wilcox.test and shown as boxplots using the ggplot function.

### 4.5. Targeted Metabolome Analysis (Lipidomic Analysis)

#### 4.5.1. Sample Preparation

Fifty fatty acid standards were properly weighed, and a concentration of 2000 μg/mL was generated for the standard linear master liquor, using dilutions with 50% acetonitrile isopropyl alcohol to create a series of concentrations of working solution. A specific concentration of isotope internal standard solution was made and mixed thoroughly to achieve the internal standard solution. The linear, internal standard, and quality control mother liquor and working solutions were preserved in the refrigerator at −20 °C. The stock solution of individual fatty acids was combined and prepared in a fatty acid-free matrix to obtain a range of fatty acid calibrators. Certain concentrations of the isotope standard were synthesized and combined as the internal standard. The stock solution and working solution were stored in a refrigerator at −20 °C. The sample size for targeted lipidomics (*n* = 3 biological replicates per stage, each comprising 100 pooled oocytes) was determined based on (1) the high technical demand and material limitations in isolating hundreds of high-quality oocytes and (2) the established methodological precedent in oocyte metabolomics, where *n* = 3 replicates is a common and published standard for identifying significant metabolic changes in already published papers [11,40,44].

#### 4.5.2. Metabolite Extraction

The samples of bovine oocytes (100 oocytes/sample, 3 samples/stage) were treated by freeze-drying and dissolved with water (100 μL). Then, it was homogenized with a mixture of 150 μL of methanol, 500 μL of MTBE, and 125 μL of water and stored at −20 °C for 30 min. The homogenized material was then centrifuged at 12,000 rpm for 10 min, and the supernatant was gathered. The extraction reagents were added to the lower layer, after which the upper organic phase was obtained. The two upper organic phases were combined and added to 500 μL of 0.4 M sodium hydroxide/methanol, and saponification proceeded at 70 °C for 30 min. The solution was subsequently cooled to room temperature and 67 μL of 3 M hydrochloric acid was added to obtain the required level of acidity. The mixture was thoroughly stirred and left to stand for 5 min. Following this, it was exposed to a centrifugal process at a speed of 12,000 rotations per minute for 10 min. Following the nitrogen blowdown, the sample was reconstituted with 100 μL of 80% methanol. The solution (50 μL) was added to the derivatization reagent (150 μL), and the derivatization procedure was carried out at 40 °C for 40 min. Then, 47.5 μL of the internal standard solution was added and completely homogenized with the sample. The final step of that procedure was the injection of the sample into the LC-MS/MS apparatus for analysis.

#### 4.5.3. LC-MS Method

An ultra-high performance liquid chromatography paired to a tandem mass spectrometry (UHPLC-MS/MS) system (ExionLC™ AD UHPLC-QTRAP 6500+; AB SCIEX Corp., Boston, MA, USA) was utilized for quantifying the fatty acids at Novogene Co., Ltd. (Beijing, China). Separation was conducted on a Waters ACQUITY UPLC BEH C18 column (2.1 × 100 mm, 1.7 μm) which was kept at 40 °C. The mobile phase, containing 0.1% formic acid in acetonitrile/water (1:1) (solvent A) and isopropanol/acetonitrile (1:1) (solvent B), was given at a flow rate of 0.30 mL/min. The solvent gradient was established as follows: initial 45% B, 1 min; 45–70% B, 4.5 min; 70–75% B, 9 min; 75–80% B, 12.5 min; 80–100% B, 14 min; 100-45% B, 15.1 min; 45% B, 17 min. The mass spectrometer was run in the negative multiple reaction mode (MRM) mode. Parameters were as follows: IonSpray Voltage (−4500 V); Curtain Gas (35 psi); Ion Source Temp (550 °C); and Ion Source Gas of 1 and 2 (60 psi).

#### 4.5.4. Quality Control

The quality control (QC) process is a critical component of the overall testing procedure, responsible for guaranteeing the consistency as well as accuracy of the results. During the machine operation, each batch of samples is placed into the QC sample at regular intervals, simplifying the assessment of their stability. The approach adopted entails determining the relative standard deviation (RSD) of all QC samples, depending on the concentration of the standard. The assessment aims at identifying the stability of the method, with an RSD value of ≤15% demonstrating the method’s stability and reliability. The recovery rates obtained in this investigation varied from 85 to 115%, showing low loss during the extraction and derivatization processes.

### 4.6. Data Visualisation

Boxplots with medians of the module scores in cell clusters were created using the ggplot2 package 3.5.2. The hinges of the boxplot are defined by the first and third quartiles (the 25th and 75th percentiles, respectively). In the context of ggplot boxplots, the upper whisker extends to the largest value, up to 1.5 times of the interquartile range (IQR), and the lowest whisker extends to the smallest value, which was, at most, 1.5 times the IQR. Outlying points are plotted. t-SNE plots and gene expression heatmaps were generated for comparison with the module score using Seurat, with log-normalized and scaled data. Figures were assembled using Adobe Illustrator 25.2.2 and all images were created by the authors using Adobe Illustrator.

Heatmaps were generated using the pheatmap package v1.0.13. Gene lists for expressed genes relevant to lipid metabolism have been established for each of the three mammalian species. Functional enrichment analysis of these lists was conducted using the DAVID bioinformatics database (https://davidbioinformatics.nih.gov/tools.jsp, accessed on 5 July 2025) [45,46]. Analyses were performed separately for each species using “Official_Gene_Symbol” identification and the appropriate species background. Significantly enriched pathways were found by relying on an adjusted *p*-value threshold of <0.05. Pathways primarily involved with lipid metabolism were selected for further investigation. Finally, the interactions between the identified genes and these major lipid pathways were displayed using Sankey and dot plots, which were generated with the SRplot web toolbox (https://www.bioinformatics.com.cn/en? *p* = 7, accessed on 5 July 2025). Icons used to build the graphical abstract for scRNA-seq analysis and lipidomic analysis were taken from the BioRender website (www.biorender.com, accessed on 5 July 2025). Correlation networks in Figures S3 and S4 were plotted using Cytoscape software v3.10.3.

### 4.7. Statistical Analysis

All statistical analyses were performed using R software (version 4.2.2) [47]. scRNA-seq data were processed and analyzed utilizing the Seurat software (v5.0.3; https://satijalab.org/seurat/) [48]. Differences in module scores, calculated using the Seurat function ‘AddModuleScore’, and in individual gene expression levels between GV and MII stages were tested using the two-sided Wilcoxon rank–sum test. The magnitude of these differences was assessed using Cohen’s d effect size [13,14], computed with the effsize R package v0.8.1 [49]. Correlation studies for gene–gene co-expression and gene–lipid integrative networks were performed using the Pearson correlation coefficient (r), obtained via the stats package in R. For targeted lipidomics, quality control was achieved by determining the RSD of internal standards, with the approach judged stable at an RSD ≤ 15%. Significant dynamic changes in lipid metabolites were indicated by a threshold of *p* < 0.05 and |log_2_FC| > 0.2. Functional enrichment analysis of gene lists was performed using the DAVID bioinformatics database [45,46], applying a significance criterion of Padj < 0.05 to identify relevant pathways.

## 5. Conclusions

This study provides the first integrated single-cell transcriptomic and targeted lipidomic map of oocyte lipid metabolism across bovine, mouse, and human species, revealing a core set of conserved genes that underpin fatty acid biosynthesis, elongation, and degradation. Despite this conservation, species vary remarkably in their temporal arrangement, with bovine and human oocyte elongation and β-oxidation occurring during maturation, whereas mouse oocytes maintain stable pathway activity. These metabolic strategies mirrored distinct evolutionary adaptations to oocyte size, lipid reservoirs, and developmental pace. Beyond improving our fundamental knowledge of reproductive metabolism, these outputs have straightforward implications for ART in offering a roadmap for optimizing ART strategies through species-tailored metabolic enhancement.

## Figures and Tables

**Table 1:** Lipid saturation composition and statistical comparison between GV and MII oocytes.

Saturation Category	GV (%)	MII (%)	*p*-Value	Effect Size (d)	Magnitude
Saturated	90.5	88.6	0.40	0.45	Moderate
Mono + diunsaturated	6.6	8	0.20	0.62	Large
Polyunsaturated	2.9	3.5	0.40	0.45	Moderate

## Data Availability

The datasets used in this study are accessible to the public. The mouse and human datasets [42] are accessible under accession number GSE197578, while Bos taurus (bovine) cells were obtained from our laboratory under accession number GSE304792. All other relevant data supporting the key findings of this study are available within the article and its Supplementary Information files or from the corresponding author upon reasonable request.

## Supplementary Materials

The following supporting information can be downloaded at: https://www.mdpi.com/article/10.3390/ijms27010397/s1.
