# Supplementary material for: Cross-Species Transcriptomic and Metabolomic Analysis Reveals Conserved and Divergent Fatty Acid Metabolic Regulatory Strategies During Mammalian Oocyte Maturation
Source: Int J Mol Sci. 2025 Dec 30;27(1):397. doi: 10.3390/ijms27010397 (PMC12785767; doi:10.3390/ijms27010397)
Supplement: Supplementary file 1 [file ijms-27-00397-s001.zip › ijms-4067606-SI.pdf]

## Supplementary Figures

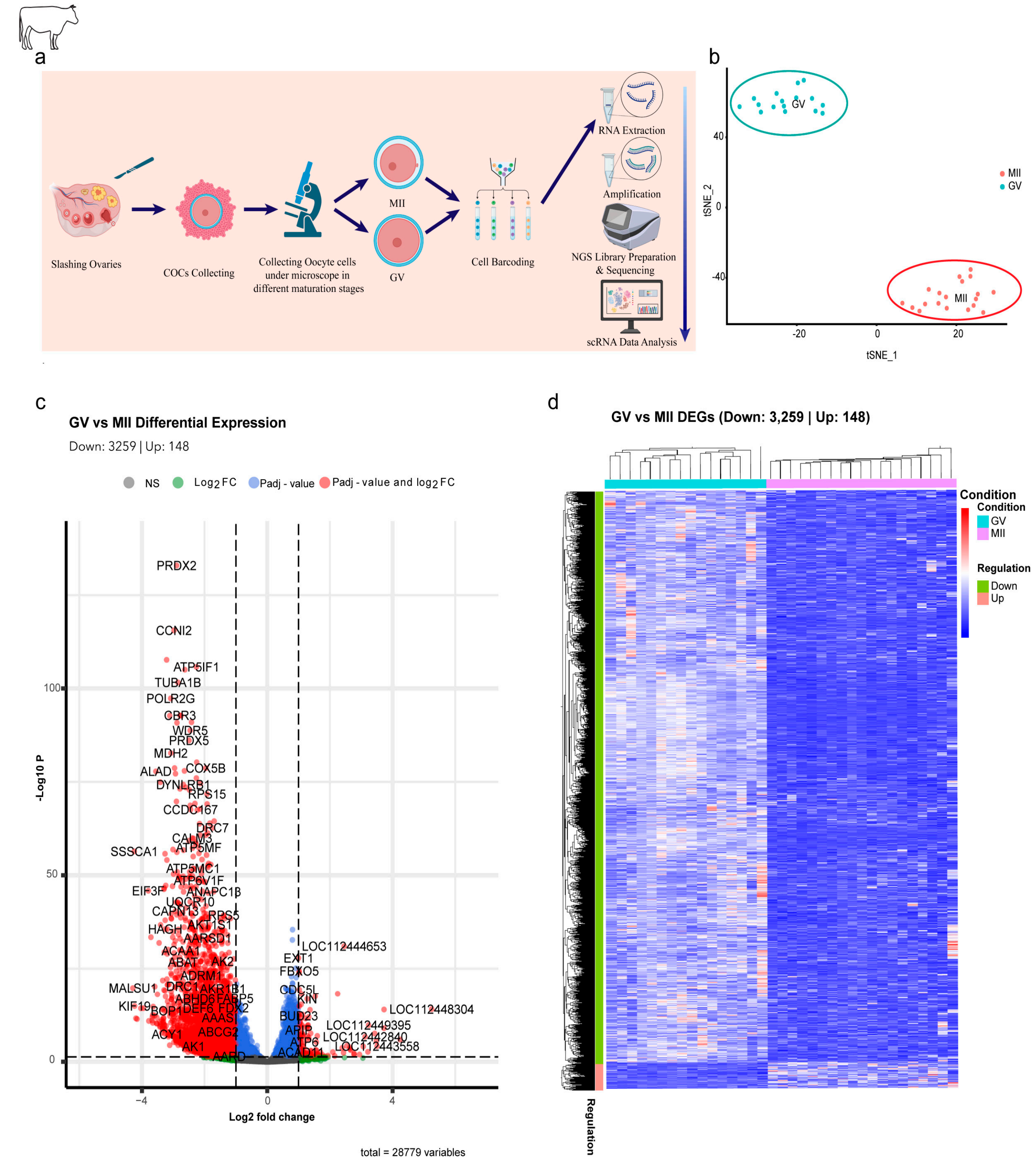


**Figure S1. Transcriptomic remodeling during bovine oocyte maturation revealed by scRNA-seq** **a.** Workflow for single-cell RNA sequencing of bovine oocytes during in vitro maturation. **b.** Single-cell transcriptome clustering by maturation stage. t-SNE plot of GV (blue) and MII (red) oocytes, demonstrating distinct transcriptional states. **c.** Volcano plot of GV vs. MII transcriptomic dynamics. DEGs (grey dots: nonsignificant; green: significant $log_2$ fold change [|FC| > 1]; blue: significant p-value [Padj < 0.05]; red: significant in both criteria). Total genes profiled:

28,779. **d.** Heatmap of significantly differentially expressed genes (DEGs) between GV and MII stages. Rows represent genes; columns represent single cells. Color scale: $log_{10}$-transformed normalized expression.

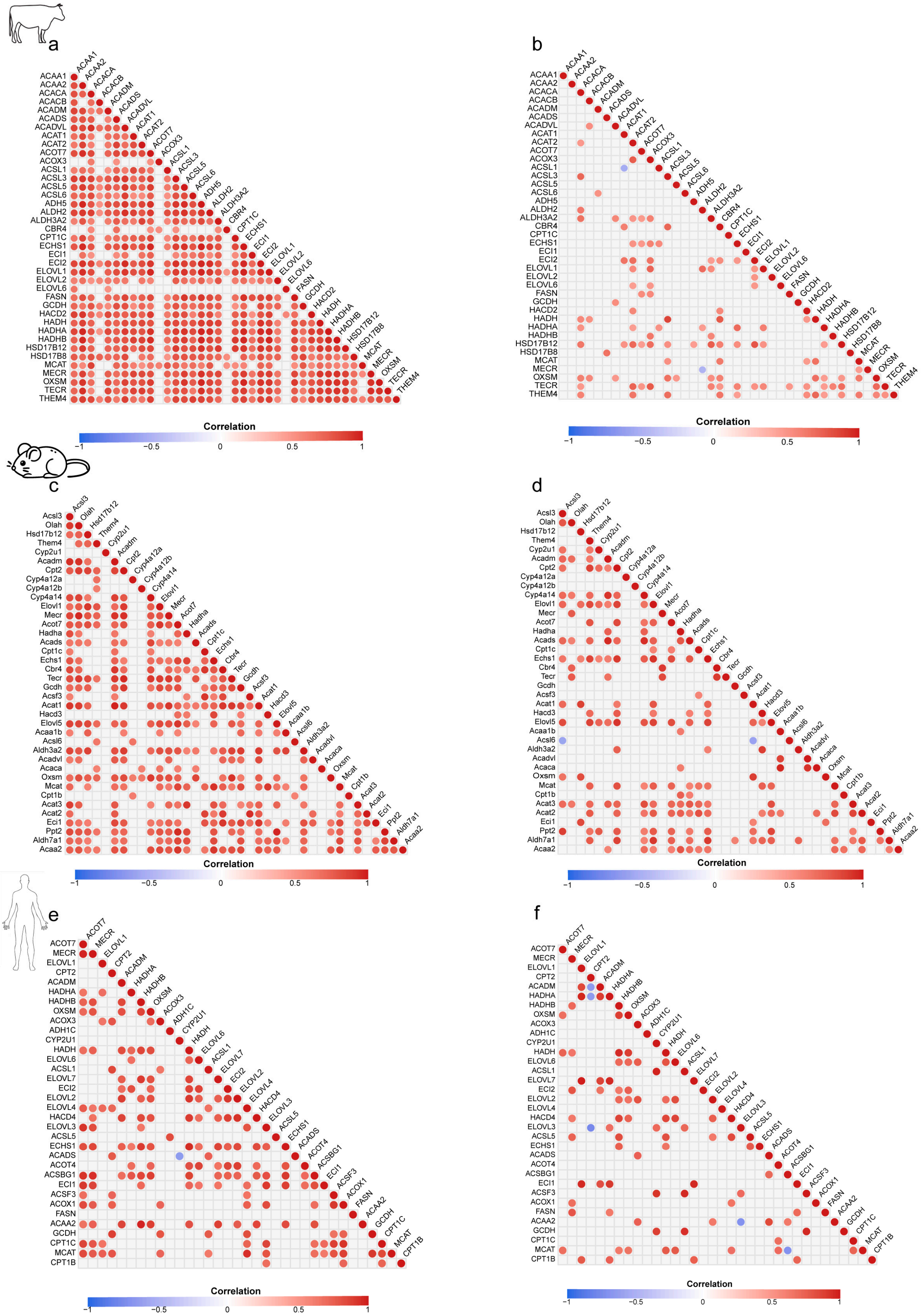


**Figure S2. Stage-specific gene co-expression networks in bovine, mouse, and human oocytes**

**a-b.** Bovine lipid metabolism gene correlations. **a.** GV stage: Strong positive correlations among all gene pairs show a unique correlation network among lipid metabolism-related genes compared to mouse and human. **b.** MII stage: Emergence of negative correlations and weaker correlations between gene pairs, suggesting catabolic/anabolic trade-offs.

**c-d.** Mouse lipid metabolism gene correlations. **c.** GV stage: Tight coupling of positive correlation. **d.** MII stage: weakened correlation with the rise of negative correlations.

**e-f.** Human lipid metabolism gene correlations. **e.** GV stage: weaker correlation compared to Bovine and Mouse, with the rise of negative correlation between *ACADS* and *CYP2U1*. **f.** MII stage: weaker compared to GV stage with 5 negative correlations raised.

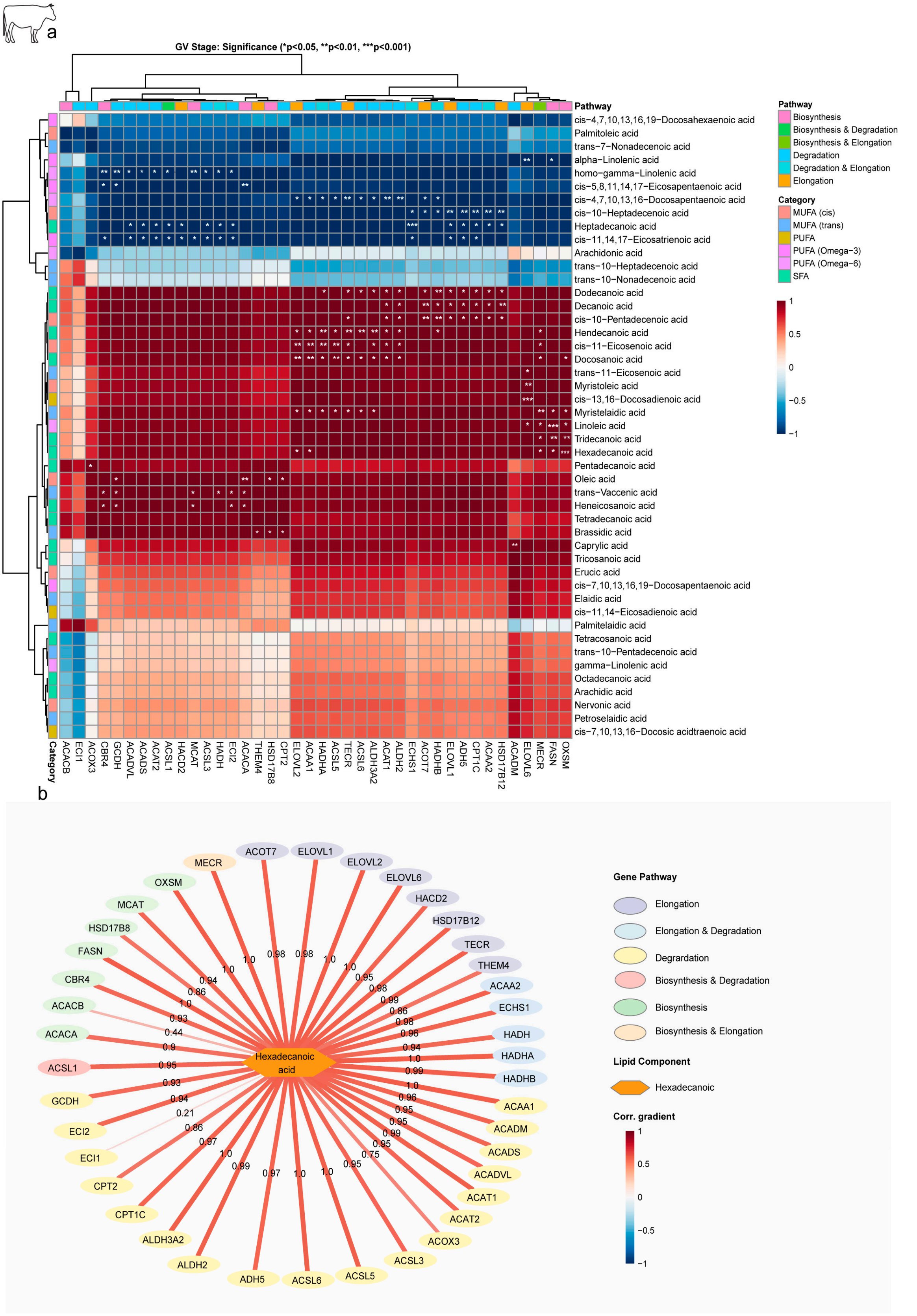


**Figure S3. Integrative network analysis links hexadecanoic acid (C16:0) to lipid metabolism genes in GV-stage oocytes**

**a.** Correlation heatmap of lipid metabolites and lipid-module genes. Rows: 47 lipid metabolites, categorized by chemical class (SFA, MUFA [cis/trans], PUFA [ω-3/ω-6]). Columns: Significant lipid-module genes, grouped by functional role: Fatty Acid Biosynthesis (FAB), Elongation (FAE), Degradation (FAD), and shred between 2 modules. Color scale: Correlation coefficient (−1 [blue, anti-correlation] to +1 [red, co-regulation]). Asterisks: Significance (*$p < 0.05$, **$p < 0.01$, ***$p < 0.001$). **b.** Network of hexadecanoic acid (C16:0) with significant lipid-module genes during GV stage. Central node: Hexadecanoic acid (hexagon), the end product of mitochondrial elongation. Gene nodes (ellipses): Color-coded by module (related to a specific module or shared between 2 modules). Edges: Correlation strength (width) and direction (color: blue = negative, red = positive).

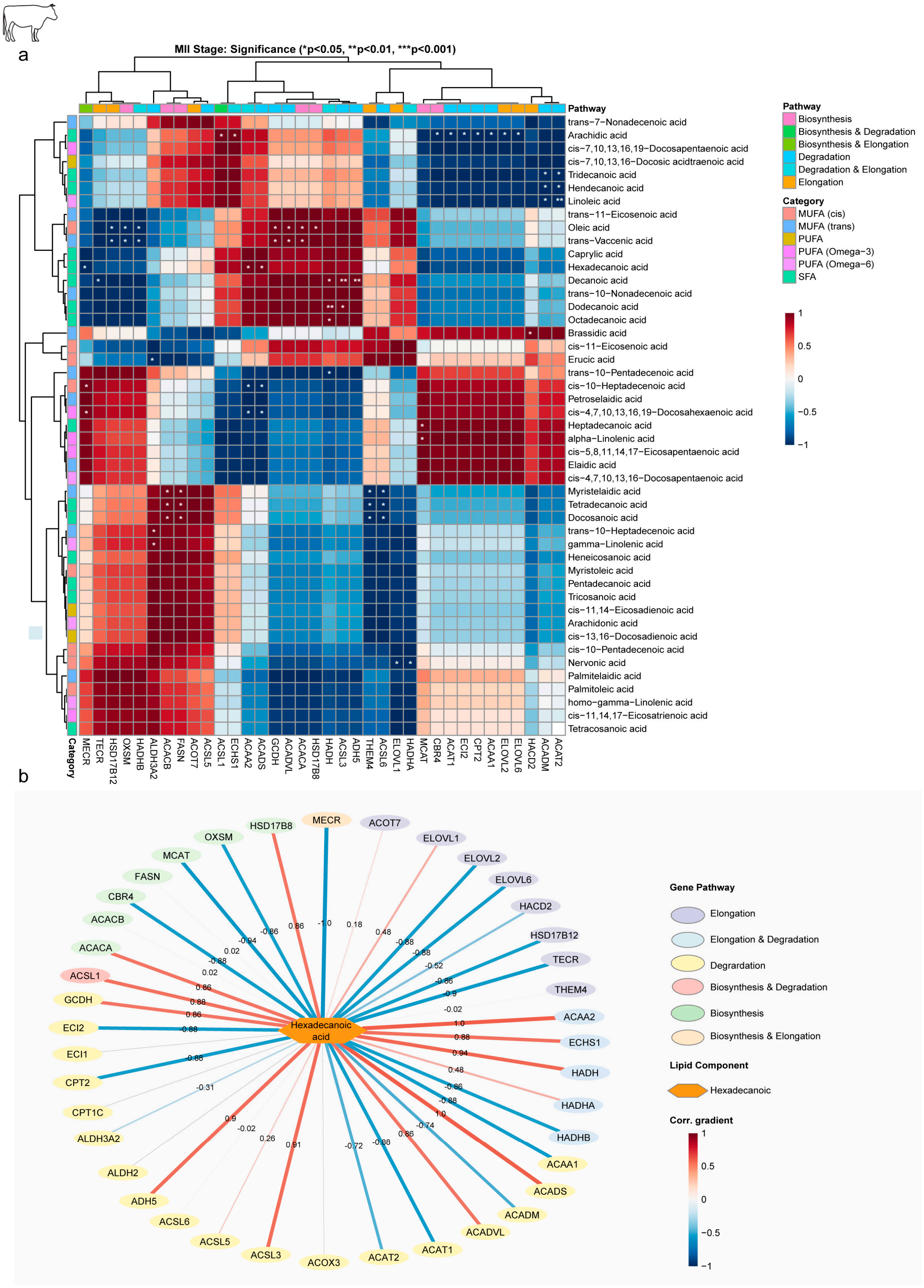


**Figure S4. Rewired correlation network of hexadecanoic acid (C16:0) with lipid metabolism genes in MII-stage oocytes**

**a.** Heatmap of metabolite-gene correlations. Rows: 47 lipid metabolites (categorized: SFA, MUFA [cis/trans], PUFA [ω-3/ω-6]). Columns: Significant lipid-module genes, grouped by pathway: Biosynthesis (FAB), Elongation (FAE), Degradation (FAD), and shared between 2 modules. Color scale: Correlation coefficient (−1 [blue] to +1 [red]). Asterisks: Significance (*$p < 0.05$, **$p < 0.01$, ***$p < 0.001$). **b.** Network of hexadecanoic acid (C16:0) interactions. Central node: Hexadecanoic acid C16:0 (hexagon), a key elongation product. Gene nodes (ellipses): Colored by module (related to a specific module or shared between 2 modules). Edges: Correlation strength/direction (blue: negative, red: positive).

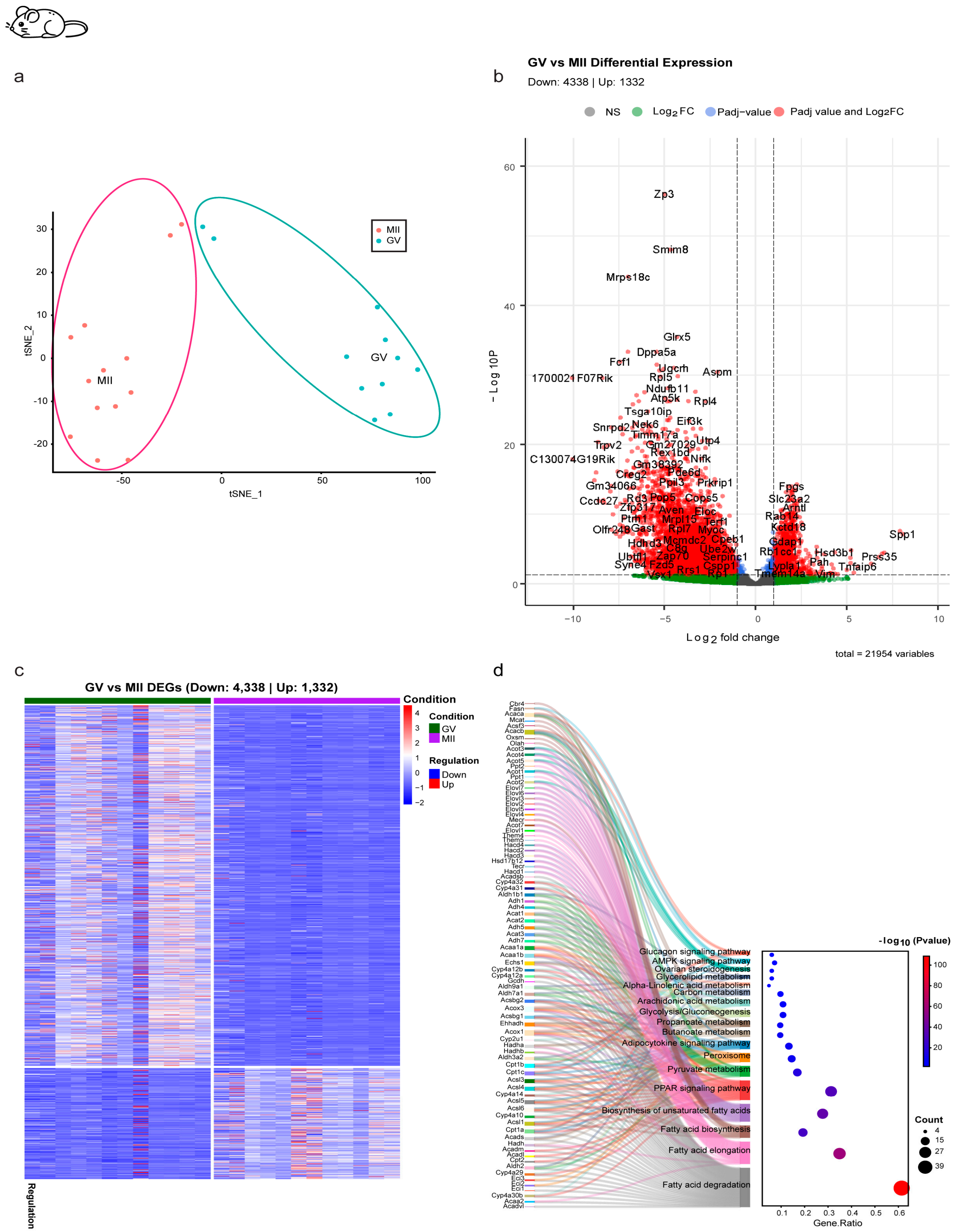


**Figure S5. Transcriptomic rewiring during mouse oocyte maturation reveals conserved and species-specific lipid metabolic shifts**

**a.** t-SNE plot of single-cell transcriptomes: Clear separation of GV (blue) and MII (red) oocytes, confirming maturation-stage-specific clustering (axes: tSNE_1/2). **b.** Volcano plot of GV vs. MII transcriptomic dynamics. DEGs (gray: nonsignificant; green: significant $\log_2$ fold change [|FC| > 1]; blue: significant p-value [Padj < 0.05]; red: significant in both criteria). Total genes profiled: 21,954. **c.** Heatmap of differentially expressed genes (DEGs) between GV and MII stages. Hierarchical clustering of 5,670 DEGs between GV and MII stages. Rows: genes; columns: single cells. Color scale: $\log_{10}$-normalized expression. **d.** Functional annotation of mouse lipid metabolism genes. Left: Sankey diagram linking genes to pathways (Fatty Acid Biosynthesis [FAB], Elongation [FAE], Degradation [FAD]). Shared genes are color-coded. Right: Dot plot of enriched pathways (GeneRatio vs. $-\log_{10}$[P-value]).

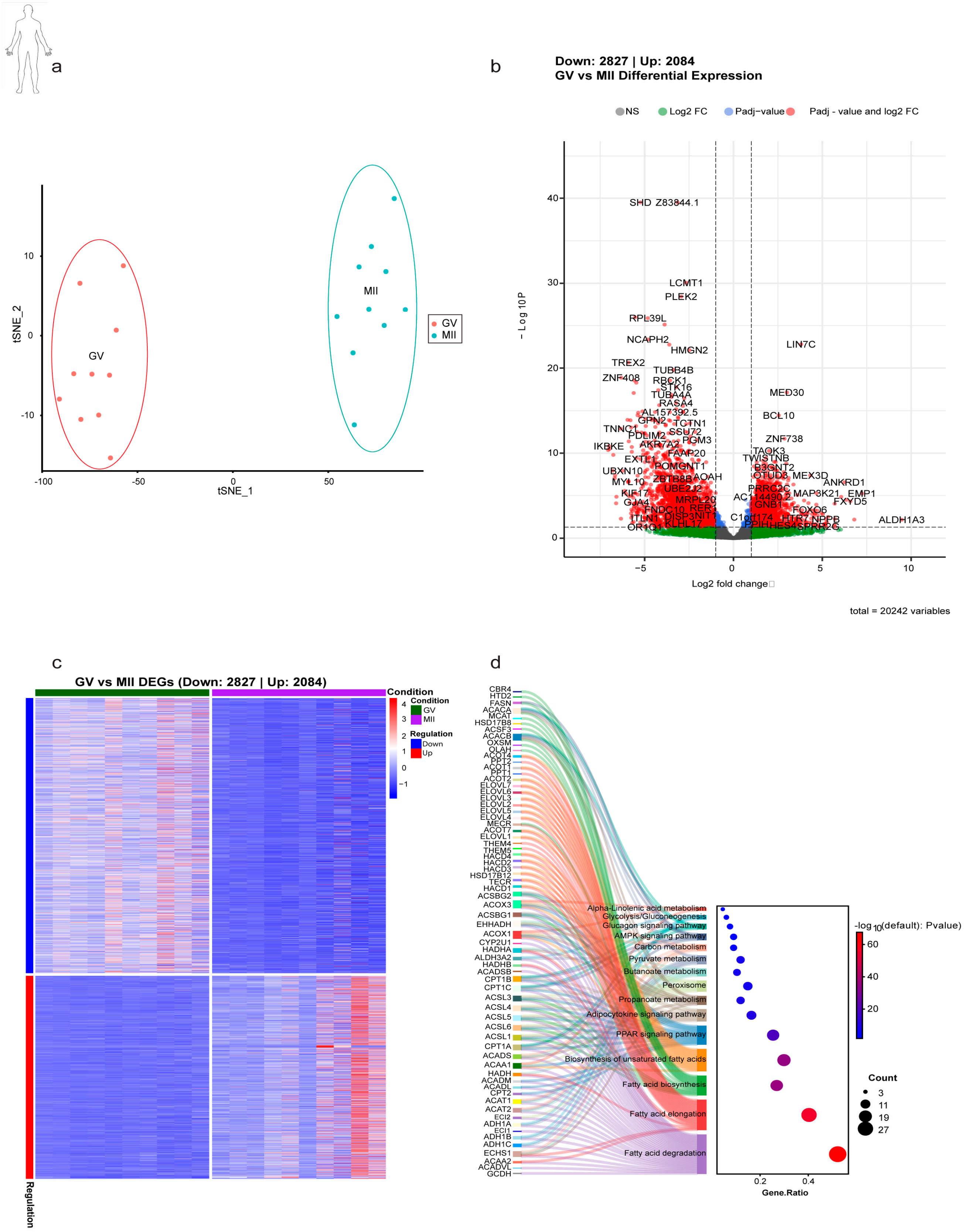


**Figure S6. Transcriptomic remodelling during human oocyte maturation reveals distinct lipid metabolic regulation**

**a.** t-SNE plot of single-cell transcriptomes. Clear separation of GV (blue) and MII (red) oocytes, confirming maturation-stage-specific clustering. **b.** Volcano plot of GV vs. MII transcriptomic dynamics. DEGs (gray: nonsignificant; green: significant $\log_2$ fold change [|FC| > 1]; blue: significant p-value [Padj < 0.05]; red: significant in both criteria). Total genes profiled: 20,242. **c.** Heatmap of differentially expressed genes (DEGs) between GV and MII stages. Hierarchical clustering of 4,911 DEGs. Rows: genes; columns: single cells. Color scale: $\log_{10}$-normalized expression. **d.** Functional annotation of human lipid metabolism genes. Left: Sankey diagram linking genes to pathways (Fatty Acid Biosynthesis [FAB], Elongation [FAE], Degradation [FAD]). Shared genes are color-coded. Right: Dot plot of enriched pathways (GeneRatio vs. $-\log_{10}$[P-value]).

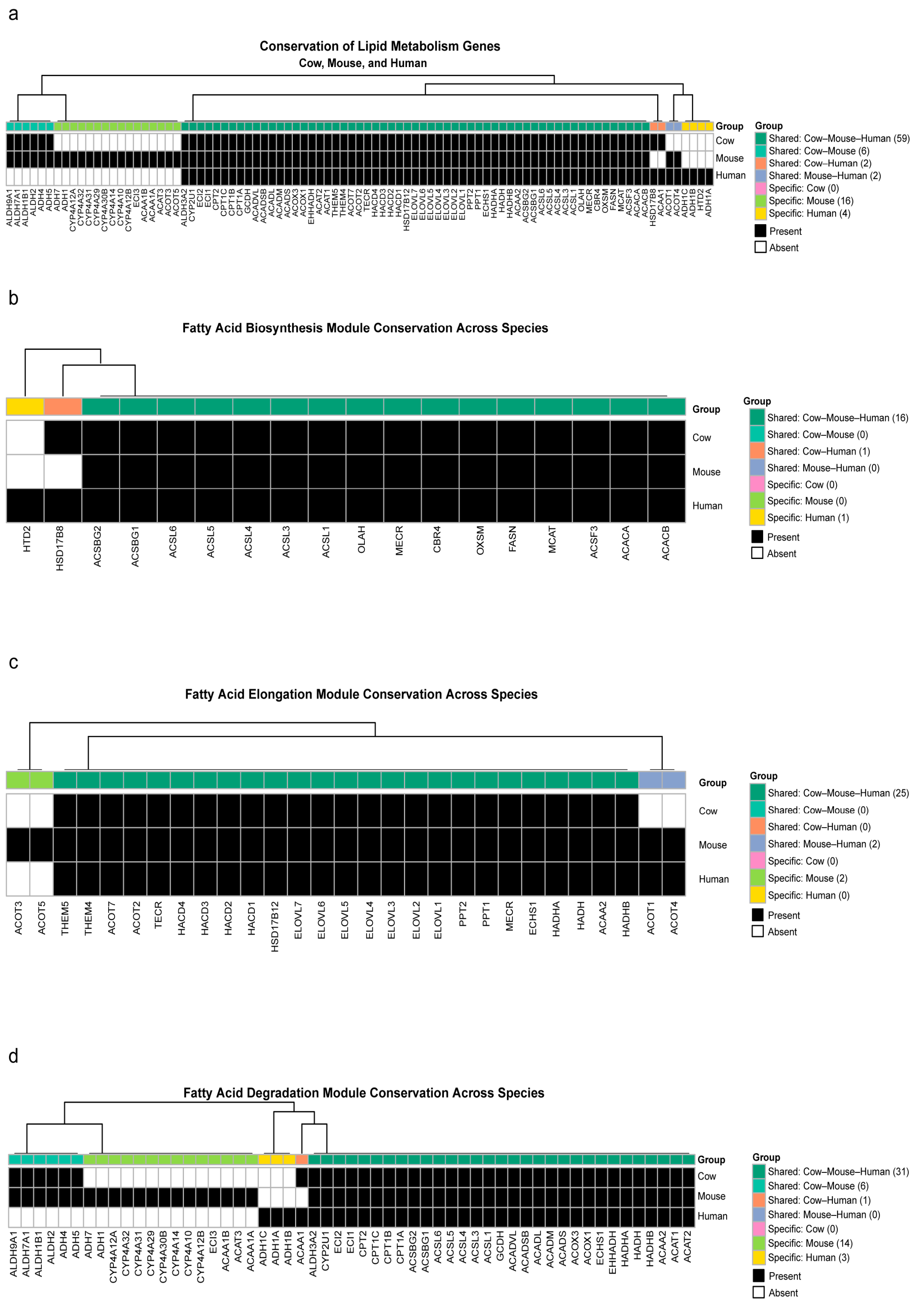


**Figure S7. Evolutionary conservation of lipid metabolism genes across bovine, mouse, and human oocytes**

**a.** Pan-species conservation of all lipid metabolism genes. Shared by all three species (59 genes). Shared between two species: Cow-Mouse (6 genes), Cow-Human (2 genes), Mouse-Human (2 genes). Species-specific genes: Human (4genes), Mouse (16genes), Bovine (0 genes). **b.** Fatty Acid Biosynthesis (FAB) module conservation. Shared Cow-Mouse-Human: 16 genes. Shared Cow-Human: 1 gene. Human-specific: *HTD2*. **c.** Fatty Acid Elongation (FAE) module conservation. 25 shared genes. Mouse–Human (2 Shared genes), Mouse-specific (2 Shared genes). **d.** Fatty Acid Degradation (FAD) module conservation 31 shared genes. Mouse-specific: (14 genes). Human-specific: (3 genes). Shared Cow-Human: (1 gene). Shared Cow–Mouse: (6 genes).

**Summary Tables of Key Results:**

Table S1: Summary of Transcriptomic and Pathway Activity Changes in Bovine Oocytes (GV vs. MII)

| Metric | GV vs. MII Outcome |
|---|---|
| Total Genes Profiled | 28,779 |
| Differentially Expressed Genes (DEGs) | 3,407 |
| DEGs Downregulated | 3,259 |
| DEGs Upregulated | 148 |
| Fatty Acid Biosynthesis (FAB) Module Score | No significant change ($p=0.42$) |
| Fatty Acid Elongation (FAE) Module Score | Significant reduction ($p=5.91\times10-9$) |
| Fatty Acid Degradation (FAD) Module Score | Significant reduction ($p=1.97\times10-9$) |

Table S2: Changes in Lipid Metabolite Abundance and Proportional Composition in Bovine Oocytes

| Lipid Metric | GV vs. MII Outcome |
|---|---|
| Significantly Downregulated Lipids | Hexadecanoic acid (*C16:0*), Octadecanoic acid (*C18:0*), Arachidic acid (*C20:0*) |
| *C16:0* Quantitative Change | 15% decrease ($p<0.001$) |
| *C18:0* Quantitative Change | 18% decrease ($p=0.019$) |
| Proportional Composition SFAs | Decrease from 90.5% to 88.6% |
| Proportional Composition MUFAs | Increase from 6.6% to 7.9% |

| Lipid Metric | GV vs. MII Outcome |
|---|---|
| Proportional Composition PUFAs | Increase from 2.9% to 3.5% |
| MUFA/SFA Ratio | 22.5% increase (effect size δ=0.62) |
| PUFA/SFA Ratio | 21% increase (effect size δ=0.45) |

Table S3: Cross-Species Comparison of Lipid Metabolism Gene Regulation

| Species | DEGs (Down/Up) | Module Score Changes (FAB, FAE, FAD) | Key Gene Dynamics |
|---|---|---|---|
| Bovine | 3,259/148 | FAB: Stable; FAE/FAD: Significant Reduction | *ACSL3* Upregulated; *CPT1C* Downregulated; *CPT2* Stable |
| Mouse | 4,338/1,332 | FAB/FAE/FAD: Stable | *Acsl3* Uniquely Upregulated; All CPT family genes Downregulated |
| Human | 2,827/2,084 | FAB/FAE/FAD: Significant Reduction | *ELOVL4* Upregulated; *CPT1C* Downregulated; *CPT2* Stable |
